# Microstructural, Mechanical, and Electrochemical Characterization of Calcium-Modified Mg-Zn Biodegradable Alloys

**DOI:** 10.3390/jfb17100485

**Published:** 2026-09-24

**Authors:** Francisco Miguel Sanchez-Sosa, Cristina Jimenez-Marcos, Julia Claudia Mirza-Rosca, Ionelia Voiculescu

**Affiliations:** 1 Mechanical Engineering Department, Las Palmas de Gran Canaria University, 35017 Tafira, Spain; francisco.sanchez119@alu.ulpgc.es (F.M.S.-S.); cristina.jimenez112@alu.ulpgc.es (C.J.-M.); 2 Materials Engineering and Welding Department, Transilvania University of Brasov, 500036 Brasov, Romania; 3 Quality Engineering and Industrial Technology Department, Industrial Engineering and Robotics Faculty, National University of Science and Technology Politehnica Bucharest, 313, Splaiul Independentei, 060042 Bucharest, Romania

**Keywords:** Mg-Ca-Zn alloys, biodegradable biomaterials, levitation furnace, microhardness, corrosion, electrochemical impedance spectroscopy

## Abstract

Magnesium has been of interest to researchers as a potential biodegradable alternative to metal orthopedic implants due to its biocompatibility and mechanical properties that are similar to those of bone. However, magnesium implants corrode more quickly than would be beneficial. This study focused on the influence of calcium modification on the microstructure and mechanical and electrochemical degradation (in simulated body fluids at 25 °C and 40 °C) of selected Mg-Zn alloys. Four alloys, Mg1Zn, Mg2Zn, Mg1Ca1Zn, and Mg1Ca2Zn, were fabricated by levitation induction melting under an argon atmosphere. Microstructural characterization was performed using optical microscopy and SEM/EDS analysis, mechanical behavior was evaluated by microhardness testing and Gaussian mixture modeling, and corrosion performance was assessed in Ringer’s lactate solution. The results indicated that calcium addition modified the microstructural morphology. Moderate Ca addition modified the distribution of secondary phases and altered the local mechanical response of the Mg matrix. Among the investigated compositions, Mg1Ca1Zn exhibited the best balance between the microstructural characteristics, mechanical properties, and corrosion resistance. These results provide useful criteria for the further development of Mg-based alloys for temporary bone fixation applications, particularly where compressive loading is predominant.

## 1. Introduction

Orthopedic implants have traditionally been manufactured from metallic materials, such as stainless steels and titanium alloys, but also from ceramic- and polymer-based composites [1,2]. However, metallic implants can release potentially toxic metal ions, and these persist in the body for long periods of time, leading to inflammatory responses or infections during long-term service [3,4,5]. Conventional polymeric and ceramic implants, on other hand, generally exhibit insufficient mechanical strength and poor load-bearing capability [6,7] compared with natural bone. Moreover, once tissue healing and regeneration are completed, a secondary surgical procedure is required to remove the permanent implants [8,9], exposing the patients to additional pain, infection risk and additional expenses. To overcome these limitations, increasing attention has been directed toward the development of biodegradable implant materials [10,11] that combine good biocompatibility, mechanical properties comparable to those of human bone, and a controlled degradation rate.

In this regard, magnesium Mg has emerged as one of the most promising candidates for the next generation of orthopedic implant materials [12,13]. Mg is a non-toxic and biocompatible element that is abundantly present in human bone, where it plays a critical role in bone metabolism, mineral homeostasis, and the regulation of bone density. Moreover, its elastic modulus and density are closer to those of natural bone compared with conventional metallic implants, which helps to minimize stress-shielding effects [14]. Despite these advantages, the practical application of pure Mg as an implant material is severely limited by its excessively rapid and uncontrolled corrosion rate under physiological conditions. During Mg degradation in physiological environments, substantial amounts of hydrogen gas are generated, which accumulates around the implant and hinders tissue healing. While pure Mg has attractive characteristics as an implant material, this option presents serious problems due to the rapid and largely unpredictable rate of corrosion in tissue systems. As Mg corrodes, H_2_ gas is formed and may accumulate to a level that compresses the surrounding tissues and compromises their integrity. The corrosion of Mg and the associated release of H_2_ gas is a major drawback for the development of Mg-based biodegradable implant systems [15]. As a result, there has been considerable work done on developing Mg-based biodegradable systems in which the corrosion of the Mg can be controlled and can be used to enhance the mechanical properties of the alloys.

Consequently, extensive research efforts have focused on developing biocompatible Mg alloys with tailored corrosion behaviors and mechanical properties. Alloying elements, such as calcium (Ca), zinc (Zn), tin (Sn), strontium (Sr), and rare earth (RE) elements, have been widely explored to improve corrosion resistance, mechanical strength, and biological performance [16]. An ideal Mg orthopedic implant alloy should therefore exhibit excellent biocompatibility, controlled biodegradability, low density, and mechanical properties compatible with human bone.

Among the various Mg alloys systems, Mg-Ca alloys have attracted significant attention for biomedical implant applications due to the excellent biocompatibility and favorable mechanical characteristics of both elements. Magnesium and calcium possess high strength-to-weight ratios, and calcium is an essential mineral naturally present in human bone while magnesium plays an important role in facilitating calcium transport and bone mineralization. In the Mg-Ca binary system, calcium exhibits a limited solid solubility in magnesium [17], with a maximum value of approximately 1.34 wt.% at 789.5 K, leading to the formation of secondary phases when this limit is exceeded. Consequently, extensive research [9,10,18] has focused on the development and optimization of Mg-Ca alloys, with systematic investigations into their microstructures, mechanical behavior, and corrosion behavior under both in vitro and in vivo conditions. Studies [17] have shown that cast Mg-xCa alloys (x = 1–3 wt.%) typically consist of an α-Mg matrix and an intermetallic Mg_2_Ca phase. As the calcium content increases, the volume fraction of Mg_2_Ca phase also increases and has been associated with accelerated corrosion due to microgalvanic coupling between phases in physiological environments [19]. Furthermore, excessive calcium addition has been reported [17,20,21] to deteriorate the mechanical properties of Mg-Ca alloys, resulting in reduced strength and ductility, thereby limiting their applicability for load-bearing orthopedic implants. Li et al. [22] studied binary Mg-Ca alloys, varying the concentration from 0.5% to 20% Ca. Increasing the calcium concentration implies a higher content of the secondary phase Mg_2_Ca, distributed predominantly at the grain boundaries. This secondary phase is brittle and reduces the ductility of Mg-Ca alloys by increasing Ca concentration, and it also influences the corrosion properties of Mg alloys. A high-volume fraction of the secondary phase Mg_2_Ca reduces the corrosion resistance of Mg-Ca alloys due to the formation of galvanic micro-cells. Therefore, the beneficial effect of Ca is expected to depend strongly on its concentration and on the morphology and distribution of the resulting secondary phases.

Mg-Zn systems have been extensively investigated because zinc is an essential trace element required for numerous biological functions and is naturally present in the human body [23,24]. In addition to its nutritional role, Zn can mitigate the detrimental effects of impurities in magnesium alloys [25], thereby improving their overall corrosion behavior. The addition of Zn enhances the castability and mechanical strength of Mg alloys and contributes to surface protection through the formation of a relatively stable passive layer [26,27]. From a materials science perspective, Zn offers several advantages when alloying with Mg. It significantly improves corrosion resistance by stabilizing the surface oxide/hydroxide film and suppressing localized corrosion phenomena under physiological conditions [18,28]. Furthermore, Zn strengthens Mg alloys via solid-solution and precipitation hardening mechanisms while also promoting grain refinement, which leads to improved mechanical performance [29]. Owing to this favorable combination of biocompatibility, enhanced corrosion resistance, and mechanical integrity, Mg-Zn alloys are regarded as promising biodegradable materials for orthopedic implant applications, effectively integrating the inherent benefits of magnesium with the strengthening and protective effects of zinc. Song et al. [30] also studied the effects of Zn on the corrosion behavior of Mg alloys. It was reported that microgalvanic effects dominated the corrosion behavior of Mg-Zn alloys, thus restricting the Zn content to levels below 5%. Above 5% Zn, a high-volume fraction of secondary Mg-Zn phases is formed, which act as cathodes, thus accelerating the corrosion of the α-Mg matrix around the Mg-Zn phases in Mg alloys.

To further enhance the performance of Mg-Zn alloys, calcium is often introduced into the Mg-Ca system as a stabilizing element to effectively regulate and slow down the corrosion rate. Previous investigations have consistently demonstrated that Mg-Ca-Zn ternary alloy systems exhibit considerable potential for biomedical applications, provided that their biodegradation behavior can be precisely controlled [31,32]. In recent years, extensive research on Mg-Ca-Zn alloys has primarily focused on the influence of Zn content on the microstructure, mechanical performance and corrosion resistance, typically while maintaining the Ca concentration at relatively low levels (approximately 0.5/1 wt.%). These studies indicate that Zn addition can refine the grain structure, improve the mechanical strength and enhance corrosion resistance by modifying secondary phase formation and surface film stability [33].

Although Mg-Zn and Mg-Ca alloys have been extensively investigated, the relationship between Ca modification, secondary-phase distribution, local mechanical heterogeneity, and electrochemical degradation in selected Mg-Zn compositions remains insufficiently clarified. In particular, a better understanding of how moderate Ca contents modify the local mechanical response of the Mg matrix and its subsequent electrochemical behavior could contribute to the development of Mg alloys with more controlled degradation. Furthermore, the effect of temperature is particularly relevant because the degradation kinetics of Mg-based biomaterials are strongly dependent on the physicochemical conditions of the surrounding aqueous environment.

Accordingly, the objective of the present study was to characterize the microstructural and local mechanical and electrochemical responses of four alloys: Mg1Zn, Mg2Zn, Mg1Ca1Zn and Mg1Ca2Zn (wt.%). The alloys were fabricated by levitation melting in a protective argon atmosphere and were comprehensively evaluated by optical microscopy, SEM/EDS and Vickers microhardness. Gaussian mixture modeling was additionally applied to the microhardness distributions to evaluate local mechanical heterogeneity. Electrochemical behavior was investigated in Ringer’s lactate solution at 25 °C and 40 °C using open circuit potential, potentiodynamic polarization and electrochemical impedance spectroscopy. The aim was to establish relationships between the composition, microstructural heterogeneity, and electrochemical degradation and identify, among the investigated compositions, the alloy providing the most favorable combinations of the measured properties. In this study, the investigated alloys are considered as potential candidates for biodegradable bone fixation applications. No specific anatomical location is proposed at this stage, as the present work evaluates the materials under unloaded in vitro conditions. The influence of site-specific mechanical loading and interfacial factors, such as surface charge-related effects on the electrochemical response and degradation behavior, will therefore require further investigation.

The design principle of the new alloys was to reduce the dissolution rate of Mg in contact with fluids in the implantation environment. It has been shown that upon reaction with water, one gram of magnesium can produce about 1.081 liters of hydrogen, which causes accelerated corrosion and an early loss of mechanical integrity of the implant due to adverse reactions through the formation of subcutaneous gas bubbles. In addition, the resulting magnesium hydroxide dissolves in water, producing a colorless alkaline solution [32]. To control the dissolution rate of magnesium–zinc alloys, 1 wt.% Ca has been added.

## 2. Materials and Methods

### 2.1. Preparation of the Mg-Ca-Zn Alloys

The Mg-Ca-Zn alloys were made in a levitation furnace from the Politehnica University of Bucharest, ERAMET laboratory (Aages, Tg. Mures, Romania), using magnesium granular materials of 2 to 5 mm in size (from MaTeek GmbH, Jülich, Germany, with a purity of 99.5%), and calcium and zinc grains of 1 to 2 mm in size, also with a purity of 99.5%. After weighing the alloy components, the granular materials were compressed using a hydraulic press at an applied pressure of approximately 343 MPa, resulting in compacted pellets with a height of 18 mm and a width of 27 mm, which weighed approximately 15 g each.

During the melting process, the following working parameters were used: current intensity (I_mf_), 83 to 85 A; melting power (P_mf_), 30.2 to 33 kW; voltage in the medium frequency range (U_mf_), 565 to 570 V; a frequency of 67 kHz; and a melting time from 2.6 to 3 min. The melted alloys were then solidified in a copper mold, producing small ingots in the form of bars about 65 mm long and 12 mm wide. Each sample was weighed to assess the oxidation losses, which resulted to be less than 2%.

Afterwards, the samples were processed by turning to remove the layers of material that had oxidized and obtain a diameter of 10 mm ± 0.1. Small disks with a thickness of 1.2 mm (±0.2 mm) were cut using an IsoMet 4000 linear precision saw (Buehler, Düsseldorf, Germany), and the surfaces were ground and polished to facilitate the study of their chemical micro-composition using the EDS method. No etching reagent was used to prevent changes in the chemical composition.

The investigated alloys were designated throughout this study as Mg1Zn, Mg2Zn, Mg1Ca1Zn and Mg1Ca2Zn, according to their nominal alloying-element contents (wt.%). The four samples were embedded in an epoxy resin–catalyst mixture and demolded after 24 h of curing. The specimens were then ground sequentially using SiC abrasive papers from P280 to P2500 grit with a Struers TegraPol-11 polishing system (Ballerup, Denmark), followed by a final polishing with a 0.3 μm α-alumina suspension until a mirror-like surface finish was obtained (Figure 1) [27,34].

Prior to the test trials, the samples were drilled to fit a screw that was welded to a cable. This cable serves as the working electrode for the electrochemical testing. The exposed area, mass and density of each sample were determined prior to testing.

### 2.2. Microstructural Characterization

Microstructural characterization was performed using optical microscopy and scanning electron microscopy. A ZEISS AxioVert.A1 MAT (ZEISS, Jena, Germany) optical microscope was used to take pictures of the samples’ surfaces at different magnifications to analyze their microstructures. Metallographic samples were etched using a 3% Nital solution, which had the composition of 3% nitric acid and 97% ethanol, for a period of 8 to 12 s.

SEM characterization was performed using a QUANTA INSPECT F 50 field emission scanning electron microscope (FEI Electron Optics B.V., Eindhoven, The Netherlands). EDS measurements were performed at three representative locations on each alloy surface to determine the local distribution of Mg, Ca, and Zn. Because EDS provides compositional rather than crystallographic information, Ca- and Zn-rich regions are referred to as secondary phases or Ca-Zn-rich phases unless their crystallographic identity is independently established. This was used to explore the samples’ microstructures.

### 2.3. Microhardness Test and Statistical Analysis

A Future Tech FM-810 microhardness tester (Kawasaki, Japan) was used to measure microhardness following the ASTM E384-22 standard [35]. Ten indentations were made on the polished surface of each sample and loaded at different points, with each measurement lasting 15 s. The load values were 1, 5, and 10 gf (HV0.001, HV0.005, and HV0.01). Using iVicky V 2.0 software, the diagonal lengths of the indentation were measured, and the Vickers microhardness values were calculated.

On the other hand, a one-dimensional Gaussian mixture model (GMM) was used to evaluate experimental data and estimate the mechanical characteristics based on the microhardness values for each combination of the alloy and applied forces. The GMM was used to identify statistically distinct populations within the hardness distribution and to estimate their relative weights, mean hardness values and standard deviations. The optimum number of Gaussian components was selected using the Bayesian Information Criterion (BIC). The GMM populations were interpreted as statistically distinguishable local mechanical responses associated with different microstructural environments. Because an individual indentation may sample more than one microstructural constituent, the GMM populations were not considered as direct phase identification measurements.

### 2.4. Electrochemical Tests

To assess corrosion behavior, the four study samples (working electrodes), with the saturated calomel electrode (SCE) and platinum (Pt) electrode, were employed. Electrochemical tests were performed in a commercial Ringer’s lactate solution, used here as a physiological electrolyte. The measurements were performed at 25° and 40 °C and repeated 3 times for each sample. Temperatures of 25 °C and 40 °C were selected to compare a reference condition at a normal temperature with a thermal stress situation associated with febrile states. Although 37 °C better represents the physiological temperature, the wider temperature range used here allows for a more sensitive evaluation of the influence of temperature on the electrochemical response and surface stability of the material. Upon immersion of each sample, the BioLogic Essential SP-150 potentiostat-galvanostat (Seyssinet-Pariset, France) was employed, using the following techniques: E_corr_ vs. time (OCP) to ascertain the open circuit potential over time, linear polarization to measure the corrosion rate values and electrochemical impedance spectroscopy (EIS) to evaluate the impedance values of the samples.

#### 2.4.1. Open Circuit Potential (OCP)

Using the EC-Lab software’s “E_corr_ vs. time” method (EC-Lab software version 9.55), the open circuit potential (OCP) of each sample was measured for thirty minutes. Data were automatically collected every 20 s, or whenever the fluctuations went above 100 mV. The OCP evolution was used to assess the stabilization behavior of the alloy–electrolyte interface prior to subsequent electrochemical characterization.

#### 2.4.2. Potentiodynamic Polarization

Potentiodynamic polarization measurements were performed over a potential range from −2.2 to −1.0 V vs. SCE, at a scan rate of 10 mV/min, with data acquisition every 0.50 s. The resulting polarization curves were analyzed to obtain the corrosion potential (E_corr_), corrosion current density (i_corr_), cathodic and anodic Tafel slopes, and corrosion rate (CR). The exposed electrode area, alloy density, and equivalent weight were incorporated into the corrosion rate calculation.

The CR was calculated according to ASTM G102-23 [36] using the corrosion current density obtained from the linear polarization measurements. The corrosion current density (i_corr_) was determined from the corrosion current (I_corr_) by considering the exposed area of each sample. The CR is expressed in mm/year and was calculated using Equation (1):

(1) $$CR=\frac{i_{corr}\cdot K\cdot EW}{d\cdot A}$$

where the constant (K) establishes the corrosion rate units (1.288·10^5^ mm g/(A cm year)), equivalent weight (EW) in g/eq, density (d) in g/cm^3^ and area (A) of each sample (in cm^2^).

The equivalent weight (EW) was calculated for each alloy composition according to ASTM G102-23 [36] using Equation (2):

(2) $$EW=\frac{1}{\sum \frac{nifi}{Wi}}$$

where Wi is the atomic weight of the ith element in the alloy, ni is its valence and fi is its mass fraction in the alloy.

Because Mg corrosion is accompanied by hydrogen evolution and dynamic surface film formation, Tafel extrapolation may not fully represent the instantaneous or long-term degradation rate.

#### 2.4.3. Electrochemical Impedance Spectroscopy (EIS)

In the electrochemical impedance spectroscopy experiment, the potentiostatic mode is used to measure impedances. A sine wave is applied around a DC potential E, which can be set to a constant value or changed based on the cell’s equilibrium potential. The measurements of impedance and phase angle were done according to ISO 16773-1-4:2016 [37]. We applied a potential of 0.00 V to the open circuit potential (OCP) at frequencies between 200 KHz and 100 mHz. Nyquist and Bode diagrams were obtained, and an electrical equivalent circuit (EEC) was used to fit the experimental data. The circuit incorporated solution resistance, constant-phase elements, resistive contributions associated with the interfacial response, and an inductive element to account for the low-frequency response observed for selected alloys. The use of constant-phase elements rather than ideal capacitors accounts phenomenologically for the non-ideal capacitive behavior associated with surface heterogeneity and corrosion product layers.

## 3. Results and Discussion

### 3.1. Microstructural Investigation

The optical microstructures of the studied alloys are presented in Figure 2. All compositions exhibit an α-Mg matrix with secondary phases whose morphology and distribution depend on the alloy composition. For Mg1Ca1Zn, relatively coarse, equiaxed α-Mg grains can be observed, whereas increasing the Zn content to 2 wt.% in Mg1Ca2Zn produces a greater number of dark features concentrated near the grain boundaries and within the interdendritic regions. Due to the low solubility of calcium in Mg (max. 0.2% by weight at 25 °C in equilibrium) for high concentrations, small, fine and dispersed particles of Mg_2_Ca are formed [38]. Low concentrations of Zn are largely dissolved in the α-Mg matrix, and the formation of ternary intermetallic compounds of the MgCaZn type is limited. Increasing the Zn concentration produces a more pronounced segregation of Ca and Zn constituents, promoting the formation of ternary intermetallic phases (Ca_2_Mg_6_Zn_3_) [39] that preferentially precipitate along the grain boundaries, thereby clearly defining them (see Figure 2b).

The microstructures of the Mg1CaxZn samples shown in Figure 2 clearly change as the Zn content increases. In the Mg1Ca1Zn alloy, a homogeneous α-Mg matrix is observed, with relatively clean grain boundaries and a low number of dark particles. This indicates that the alloying elements are more uniformly distributed. When the Zn content increases to 2 wt.% (Mg1Ca2Zn), darker areas begin to appear at the grain boundaries with finer particles in the matrix, suggesting that some Ca and Zn are concentrated in these regions during solidification.

A comparison of the Ca-free microstructures (Figure 2a,b) and of Ca-containing alloys (Figure 2c,d) indicates that Ca modifies the morphology and spatial distribution of the secondary phases. It results in a more clearly delineated and apparently coarser grain structure compared with the binary SEM images and spectra of Figure 3, confirming and detailing the characteristics previously observed in optical metallography.

The SEM observations in Figure 3 provide higher resolution evidence of the compositional heterogeneity. Bright regions are enriched in Ca and Zn relative to the surrounding Mg matrix, and their fraction and spatial continuity increase with the alloy composition.

In the sample with the lowest Zn content, a relatively continuous Mg matrix can be distinguished, in which small, bright particles appear scattered, associated with phases rich in Ca and Zn. As the Zn content increases, these phases become increasingly concentrated in the interdendritic zones and grain boundaries, forming a more defined network. In alloys with higher Zn contents, this network becomes more continuous and is consistent with an eutectic or interdendritic secondary constituent rich in Ca and Zn, which is formed during solidification through their segregation and accumulation from the liquid alloy. Based on the alloy composition and reported Mg-Ca-Zn phase diagrams, these regions are consistent with Mg_2_Ca and/or Ca_2_Mg_6_Zn_3_ intermetallic products. However, its eMgxact crystallographic identity cannot be established from SEM/EDS alone. In addition, a greater number of fine precipitates are observed within the Mg matrix, indicating that portions of Ca and Zn also precipitate from the solid solution during cooling. Overall, SEM analysis allows for clearer visualization of the distribution of these phases and confirms the evolution of the microstructure with increasing Zn content.

To evaluate the chemical composition of the samples, the procedure described in Figure 4 was followed, where z1, z2, and z3 are zones on the same surface.

EDS quantification summarizes the mean chemical compositions of the alloys under study (see Table 1). The presence and distribution of these phases in the SEM images correspond to the results of the EDS analysis, which highlights bright areas rich in Ca and Zn, suggesting the possible presence of Mg-Ca-Zn intermetallic compounds.

### 3.2. Microhardness Test

Analysis of the microhardness results obtained for the studied alloys allows the evaluation of the heterogeneity of their microstructures and mechanical behaviors under various loading conditions, which are essential aspects for assessing their potential use in temporary bone fixation applications [40]. Figure 5 presents the average microhardness for each load (1, 5, and 10 gf).

Figure 6 shows the histograms and curves of the microhardness density for each load (1, 5, and 10 gf). Also, GMM fitting is considered for each load.

The Gaussian mixture model breaks the experimental distribution down into separate mechanical populations, and a very important stage in the procedure is figuring out the average weight of each component of each group. After we have obtained the mean and standard deviation for each part, we can find the best number of components (“Comp.” column in Table 2) using the Bayesian Information Criterion (BIC).

The model almost always converges toward K = 2 (column K in Table 2, the number of components) main components. The only time this does not happen is when Mg1Ca1Zn has a 1 gf value, in which case, a third component is found.

Using the GMM made it easier to identify different microstructural constituents based on how they responded to identification. In Table 2, two main zones were identified in the formation of the groups. Component 1 from Table 2, which has the highest statistical weight, was associated with the α-Mg matrix, while the Component 2 from Table 2 corresponded to long-lasting intermetallic phases, such as Mg_2_Ca or ternary compounds of Mg-Zn-Ca (proposed by composition and the literature), which precipitated preferentially in the grains boundaries [17].

An increase in Zn from 1% to 2% would act as a reinforcement agent through solidification and dispersion. This was demonstrated by the increase in the average hardness of the secondary phases (from 0.759 GPa to 0.797 GPa under 5 gf), improving the initial mechanical integrity required for the structural support (see Table 2).

The model identified a third zone of intermediate mechanical response (0.619 GPa) in the Mg1Ca1Zn mixture under an ultra-low load (1 gf). It is highly likely that this value is an interface or boundary effect rather than a distinct physical phase (see Figure 6). The volume of deformation is small for such small loads. If the indentation occurs between a blank matrix and a harder precipitate, the recorded value is promise-weighted by the volume of both phases [17]. Because only ten indentations were obtained for each alloy and load, the GMM parameters, especially for the third component identified in Mg1Ca1Zn at 1 gf, should be regarded as exploratory rather than definitive. The component with a weight of 0.1 is supported by very limited observations and cannot independently establish a separate mechanical population; it is a tentative indication requiring spatially correlated validation.

According to ASTM E384 [35], the results for very low loads (25 gf) are typically regarded as qualitative because of the difficulty of optical measurement and the extreme sensitivity to local variations [5,7].

The microhardness study indicates that the inclusion of Ca in Mg-Zn alloys results in a notable variation in the distribution of local mechanical properties. The Mg1Ca1Zn alloy exhibits multimodal behavior (K = 3), with three distinct hardness zones detected. This means that the alloy is microstructurally quite heterogeneous due to the presence of several coexisting phases. With higher Zn contents (Mg1Ca2Zn), however, the microhardness distributions shift toward one-dimensional behavior (K = 1), although the variation in concentrations is smaller.

These results suggest that Ca initially causes a higher degree of microstructural complexity; however, as Zn concentrations increase, this leads to greater uniformity in the phase distribution and stabilizes the material’s mechanical response.

Figure 7 depicts the situations in which an indenter encounters a multifaceted microstructure.

Mg1Ca2Zn exhibits a single dominant population at all three loads, indicating that, at the spatial scale sampled by the indentation, the hardness distribution is less clearly multimodal than that of Mg1Ca1Zn. This observation should not be interpreted as proof of a more homogeneous phase distribution of hardness instead of directly resolving the underlying microstructure.

The GMM analysis demonstrates that the addition of Ca modifies the distribution of local mechanical responses in the investigated Mg-Zn alloys. The results are therefore complementary to the SEM and EDS observations and provide evidence that alloy composition influences not only the mean hardness but also the spatial heterogeneity of the mechanical response.

### 3.3. Electrochemical Tests

#### 3.3.1. Open Circuit Potential

Figure 8 presents the variation in open circuit potential (OCP) as a function of immersion time for MgxZn and Mg1CaxZn (x = 1, 2) alloys, measured at 25 °C and 40 °C, respectively.

It can be observed that, at 40 °C, all alloys exhibited more negative potential OCP values compared to 25 °C, reflecting accelerated reaction kinetics at elevated temperatures. Over time, the OCP values of all alloys gradually shifted toward more positive potentials, suggesting an establishment of a dynamic equilibrium between the film formation and its dissolution. The more negative OCP values observed at 40 °C indicated a change due to the temperature of the interfacial electrochemical state. This behavior is consistent with previous studies showing that the temperature accelerates the corrosion processes in magnesium alloys [27].

#### 3.3.2. Linear Polarization

Figure 9 shows the potentiodynamic curves for MgxZn and MgxZn1Ca (x = 1, 2) alloys, measured at 25 °C and 40 °C, while Table 3 provides the electrochemical parameters derived from the Tafel fit.

A rise in temperature from 25 °C to 40 °C has a significant effect on the electrochemical response of all alloys. In general, a shift in corrosion potential (E_corr_) towards more negative values was observed, along with a significant increase in the corrosion current density (i_corr_). Thus, the rise in temperature accelerated both anodic and cathodic reactions, which explains the significant increase in the corrosion rate (CR).

The temperature sensitivity depended strongly on the alloy composition (see Table 3). Between 25 °C and 40 °C, the corrosion rate increased by approximately 90% for Mg1Zn, 17% for Mg2Zn, 138% for Mg1Ca1Zn, and 163% for Mg1Ca2Zn. The particularly large increase observed for Mg1Ca2Zn suggests that its continuous secondary phase network rich in Ca and Zn may promote a stronger microgalvanic coupling and a greater disruption of the surface layer at elevated temperatures. These results indicate that the effect of temperature is strongly modulated by the alloy composition and microstructural characteristics rather than being solely a general kinetic effect.

The increase in the corrosion rate observed at 40 °C compared to 25 °C can be elucidated by the interplay of enhanced electrochemical kinetics and intensified mass transport within the electrolyte. In this work, this second effect is discussed using the Stokes–Einstein equation, which shows that as the viscosity of the medium drops from 0.89 mPa·s (25 °C) to 0.65 mPa·s (40 °C), the term proportional to the diffusion T/η rises from 335 to 481.7.

The Stokes–Einstein relation [41] provides a qualitative framework for discussing the possible influence of temperature-dependent viscosity on diffusional mass transport in the electrolyte:

(3) $$D=\frac{k\cdot T}{\left(C\cdot \eta \cdot r\right)}\approx \frac{T}{\eta }$$

where D is the diffusion coefficient, k is the Boltzmann constant, T is the temperature, °C is a constant depending on the interface, r is the radius of the macroscopic particle and η is the viscosity of the fluid [41].

The approximate viscosity values of water used in this analysis are η_1_ = 0.89 mPa·s at 298.15 K (25 °C) and η_2_ = 0.65 mPa·s at 313.15 K (40 °C). The relative decrease in viscosity from 25 °C to 40 °C is 27%, and the relative increase in the diffusion coefficient is 43.8%, values which should be regarded as a qualitative estimate of the possible mass transport contribution.

This link between diffusion, temperature, and viscosity fits with the widely accepted theoretical framework for diffusive systems (Einstein, 1905). So, the rise in D makes it easier for aggressive species (mostly Cl^−^) to get to the metal–electrolyte interface and speeds up the removal of reaction products, which makes the boundary layer less of a problem. This increase in mass transport is particularly significant in Mg alloys within chloride-rich environments, as chloride destabilizes the Mg(OH)_2_-based film, resulting in dissolution–reformation cycles that lack protective qualities, as demonstrated in classical studies on the dissolution of Mg in the presence of halides [42].

In line with this framework, the polarization results of this article demonstrate a systematic increase in i_corr_ and CR when the temperature is raised from 25 °C to 40 °C for all the studied alloys. This trend aligns with experimental findings regarding biodegradable Mg alloys in physiological solutions, where elevated temperatures significantly accelerate their degradation [43].

EIS fitting also shows that the resistive contributions at the interface decrease with temperature, meaning that the film is less protective and the interface is more electrochemically active at high temperatures.

Finally, it should be noted that the response to temperature is modulated by the microstructure, as the presence and distribution of secondary phases can induce microgalvanic couplings and promote localized attack in Mg alloys, as previously described for Mg systems, thereby reinforcing the link between microstructural heterogeneity and accelerated degradation [44].

The observed increase in the corrosion rate with the temperature is due to the combined effect of enhanced electrochemical kinetics, altered surface film stability and temperature-dependent mass transport.

#### 3.3.3. Electrochemical Impedance Spectroscopy

Electrochemical impedance spectroscopy (EIS) is a non-destructive and highly sensitive technique that enables the real-time monitoring of corrosion processes without altering the metal surface. It separates and quantifies the electrochemical processes, permitting a detailed analysis of corrosion mechanisms.

Figure 10 presents the Nyquist plots of the MgxZn and MgxZn1Ca alloys measured in Ringer’s solution at 25 °C. In the high-frequency region, the intercept close to the origin reflects the solution resistance (R_s_), and it can be observed that all alloys showed similar R_s_, meaning that the electrolyte resistance was comparable for all tests. The diameter of the semicircle is mainly associated with the charge-transfer resistance (R_ct_), and the larger the diameter of the semicircle, the higher R_ct_, which means that resistance to corrosion is also higher. Mg1Zn showed a higher semicircle (extending to 2500–3000 Ω·cm^2^), closely followed by Mg1Ca1Zn, while the other three alloys showed smaller semicircles, ending near 500–1000 Ω·cm^2^. Mg1Zn exhibited the highest fitted resistance to electrochemical charge transfer and the largest impedance response at 25 °C, indicating a highly resistive interfacial film under EIS conditions. However, Mg1Ca1Zn had the lowest corrosion rate from the polarization data, indicating the most favorable result according to the potentiodynamic measurements. These results demonstrate that the two techniques provide complementary rather than identical rankings of electrochemical performance. It can be observed that the arcs are not perfect semicircles, which indicates that the surface heterogeneity of the alloys has a non-ideal capacitive behavior; this is why a constant-phase element will be used in the equivalent electrical circuit instead of an ideal capacitor. In the low-frequency region, a tail can be observed for Mg1Zn and Mg1Ca1Zn, suggesting a possible diffusion process.

For Mg2Zn and Mg1Ca2Zn alloys, different behaviors can be observed in the low-frequencies region. In this region, both alloys exhibited a deviation from ideal capacitive behavior, manifested by a downturn of the impedance toward negative imaginary values. This behavior is commonly associated with inductive or relaxation phenomena, which are attributed to the adsorption/desorption of intermediate species, such as Mg^2+^, Ca^2+^ or Zn^2+^, or to the partial breakdown and repair of the surface corrosion film.

The inductive loop appearing at low frequencies in the alloys with higher concentrations of Zn, with and without Ca additions, suggests the adsorption of intermediate Mg^+^ species, indicating that Ca additions increase electrochemical activity when excessive secondary phases are formed.

The Bode plots (see Figure 11 and Figure 12) confirm that Mg1Zn exhibits superior corrosion resistance, as evidenced by its higher low-frequency impedance modulus and larger phase angle. Increasing Zn contents decrease the impedance and phase angle, indicating the reduced protection of the surface film and accelerated electrochemical activity.

Figure 13 shows the equivalent electrical circuit used to fit the electrochemical impedance spectroscopy (EIS) data, represented as R(CPE(R(CPE(RL)))).

This model describes the electrochemical responses of magnesium alloys in Ringer’s solution, taking into account the contribution of the solution resistance (R_s_), a porous surface layer (represented by R_P_ and CPE_P_), and a more compact internal layer (modeling using R_C_, CPE_C_, and an L_C_-inductive element). The presence of constant-phase elements (CPEs), rather than ideal capacitors, accounts for the non-ideal capacitive behavior associated with surface heterogeneity and the roughness of the corrosion product layers. The impedance expressions corresponding to the elements of the equivalent electrical circuit were calculated according to [45] as follows:

(4) $$Z_{R}=R\;\;Z_{CPE}=\frac{1}{Y^{0}{\left(jw\right)}^{n}}\;Z_{L}=jw$$

(5) $$Z_{CPERL}={\left(Y_{C}^{0}{\left(jw\right)}^{n_{C}}+\frac{1}{R_{C}+jwL}\right)}^{-1}$$

(6) $$Z_{R(CPERL)}=R_{p}+{\left(Y_{C}^{0}{\left(jw\right)}^{n_{C}}+\frac{1}{R_{C}+jwL}\right)}^{-1}$$

(7) $$Z_{CPE(R\left(QRL\right))}={\left(Y_{P}^{0}{\left(jw\right)}^{n_{P}}+\frac{1}{R_{P}+{\left(Y_{C}^{0}{\left(jw\right)}^{n_{C}}+\frac{1}{R_{C}+jwL}\right)}^{-1}}\right)}^{-1}$$

(8) $$Z\left(f\right)=R_{s}+{\left(Y_{P}^{0}{\left(jw\right)}^{n_{P}}+\frac{1}{R_{P}+{\left(Y_{C}^{0}{\left(jw\right)}^{n_{C}}+\frac{1}{R_{C}+jwL}\right)}^{-1}}\right)}^{-1}$$

As shown in Table 4, the results of the impedance-fitted data suggest that the total corrosion resistance (R_T_ = R_P_ + R_C_) depend on the temperature and alloy composition. At 25 °C and at 40 °C, the Mg1Zn and Mg1Ca1Zn alloys exhibited the highest R_T_ values, indicating that they offer the greatest corrosion resistance. This is consistent with the lower corrosion current densities (i_corr_) obtained from potentiodynamic tests. However, alloys with a higher Zn concentration, such as Mg1Ca2Zn, have considerably lower R_T_ values, suggesting that these surface layers offer poorer protection.

## 4. Conclusions

The present study evaluated the effects of Ca addition and temperature on the microstructure, local mechanical response, and electrochemical behavior of Mg-Zn alloys. The main conclusions are:

- Alloys from MgCaZn system can be successfully obtained using an induction melting furnace with a levitation effect, provided an inert argon protective environment is used to reduce oxidation effects.
- The addition of calcium significantly modifies Zn and the morphology and spatial distribution of secondary phases, particularly near the grain boundaries and interdendritic areas.
- The Mg1Ca1Zn alloy exhibited the greatest microstructural heterogeneity with a multimodal hardness distribution, and the GMM analysis confirmed the coexistence of multiple mechanical populations regarded as microstructure-correlated mechanical responses.
- Increasing the temperature from 25 °C to 40 °C increased the corrosion rates of alloys, with the largest increase observed for Mg1Ca2Zn.
- Mg1Zn showed the highest fitted EIS resistance at 25 °C, whereas Mg1Ca1Zn showed the lowest corrosion rate derived from the potentiodynamic data.
- The results demonstrate that controlled calcium addition represents an effective approach for tailoring the microstructure, mechanical properties, and corrosion performance of biodegradable Mg-Zn alloys. Therefore, Mg-Ca-Zn systems, particularly Mg1Ca1Zn, show promising potential for further investigation in biodegradable bone fixation applications.

Future studies will include macroscopic mechanical assessments to understand the characterization of the alloys’ mechanical properties. This will include deformation studies through tensile and compressive failures and examination of long-term immersion degradation. Additionally, in vitro cytocompatibility will be assessed. Since the alloys were examined as cast, the influence of compositional segregation on the uniformity of corrosion and the possible effects of homogenization heat treatments on the microstructure and behavior of degradation will be examined.

## Figures and Tables

**Figure 1:**
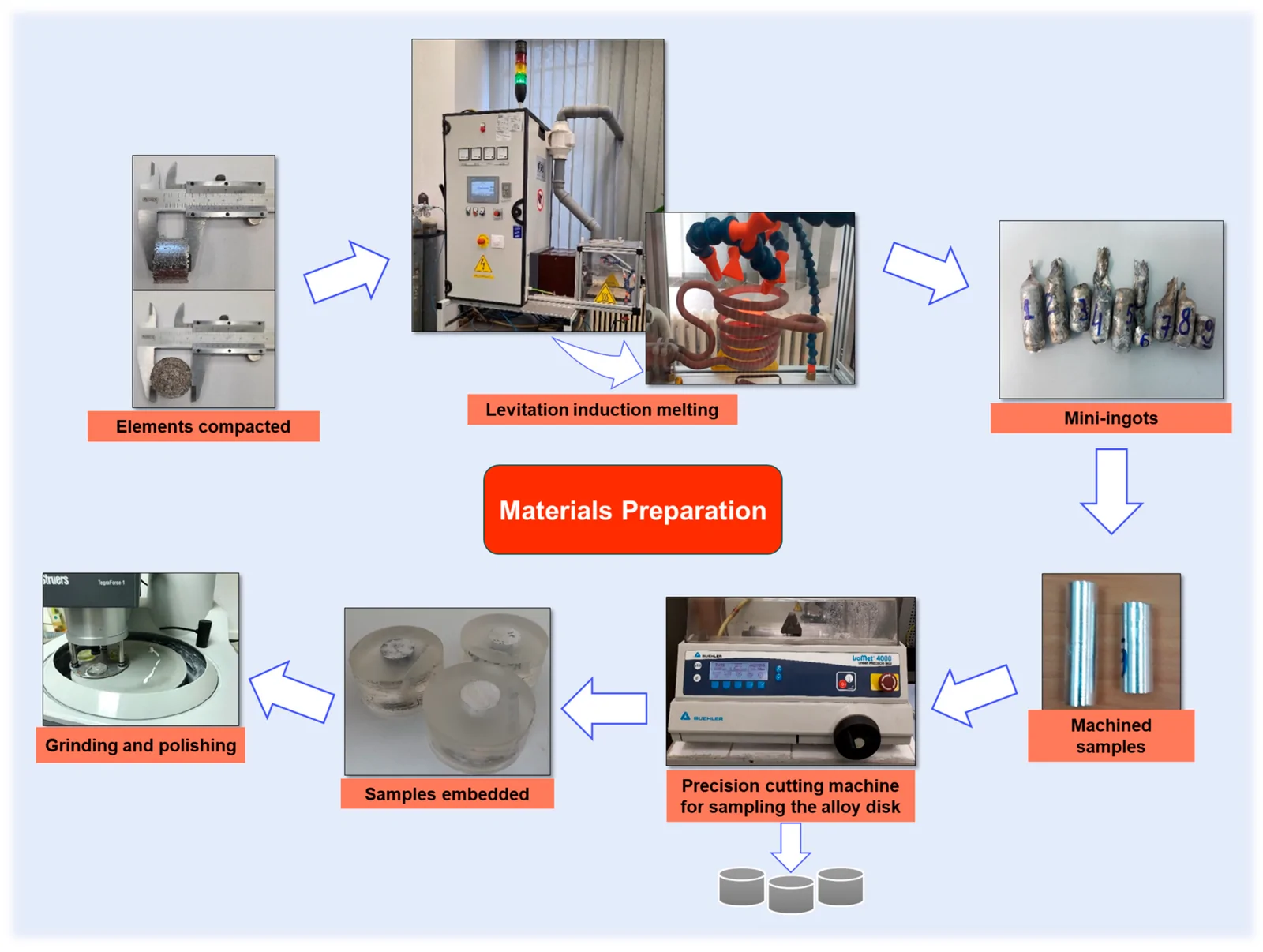
Manufactured and preparation of the MgxZn and Mg1CaxZn samples, from compacted pellets to polished samples.

**Figure 2:**
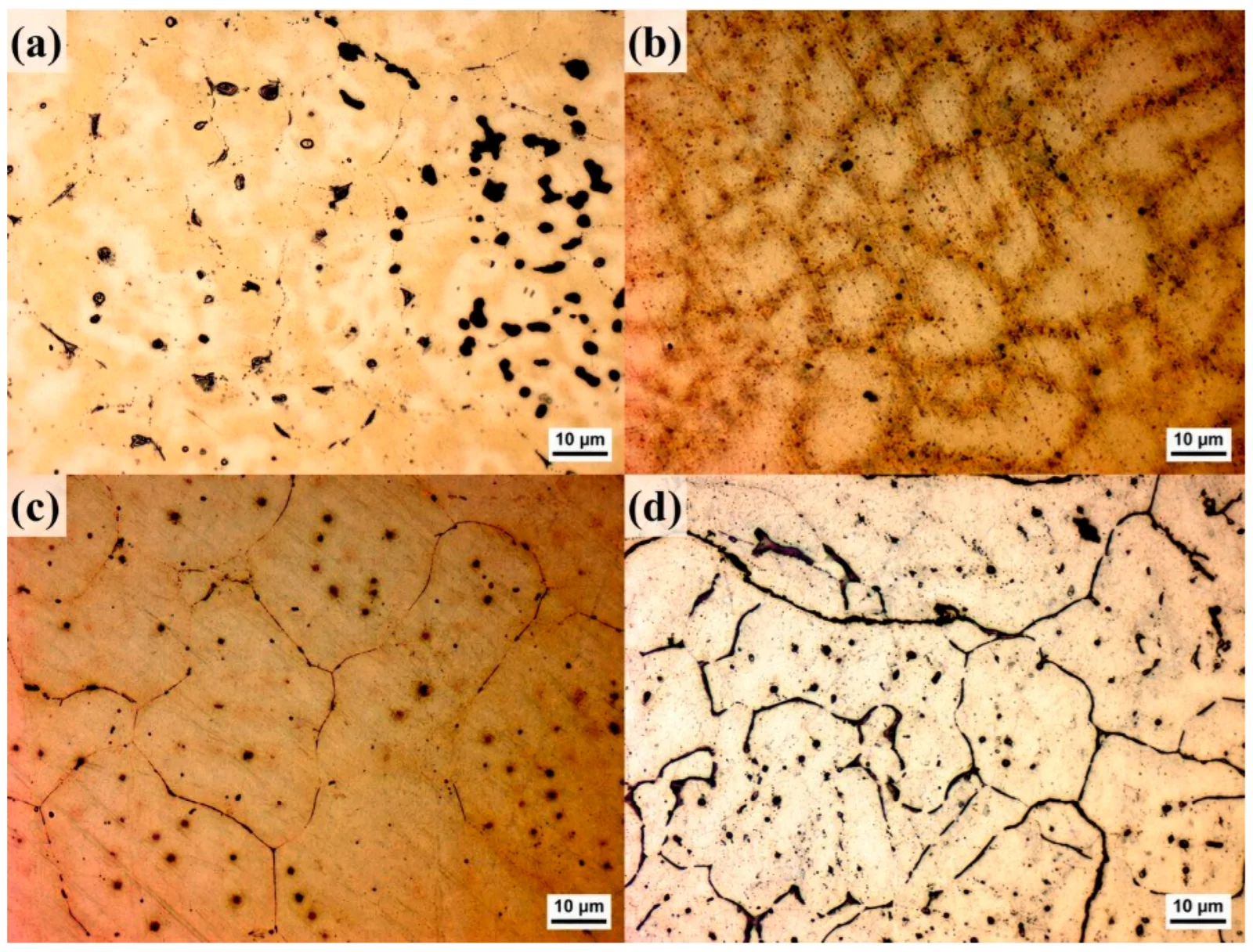
Optical microstructures of (**a**) Mg1Zn, (**b**) Mg2Zn, (**c**) Mg1Ca1Zn, and (**d**) Mg1Ca2Zn, showing the evolution of the α-Mg matrix and secondary phase morphology.

**Figure 3:**
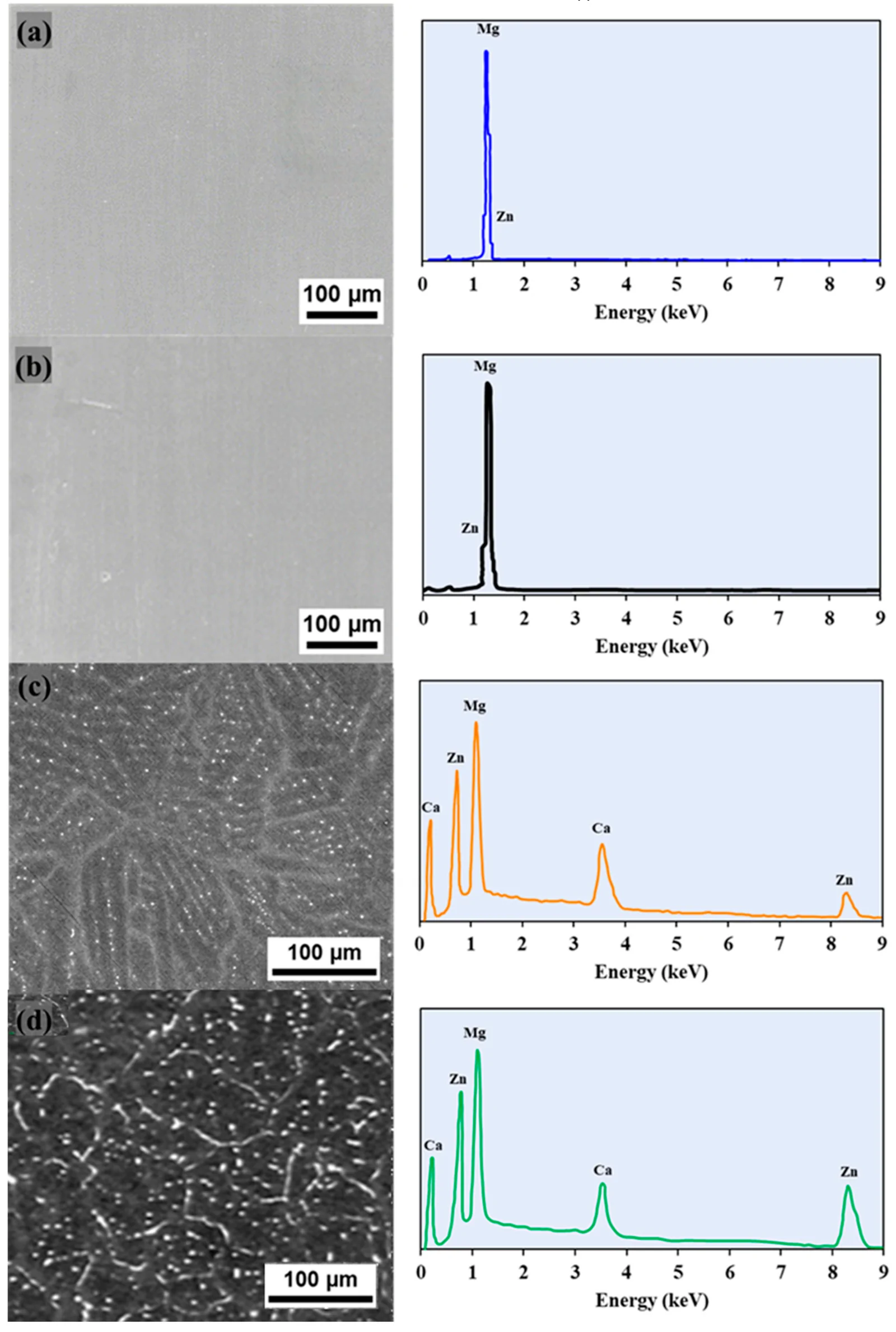
SEM images and EDX spectra of (**a**) Mg1Zn, (**b**) Mg2Zn, (**c**) Mg1Ca1Zn, and (**d**) Mg1Ca2Zn.

**Figure 4:**
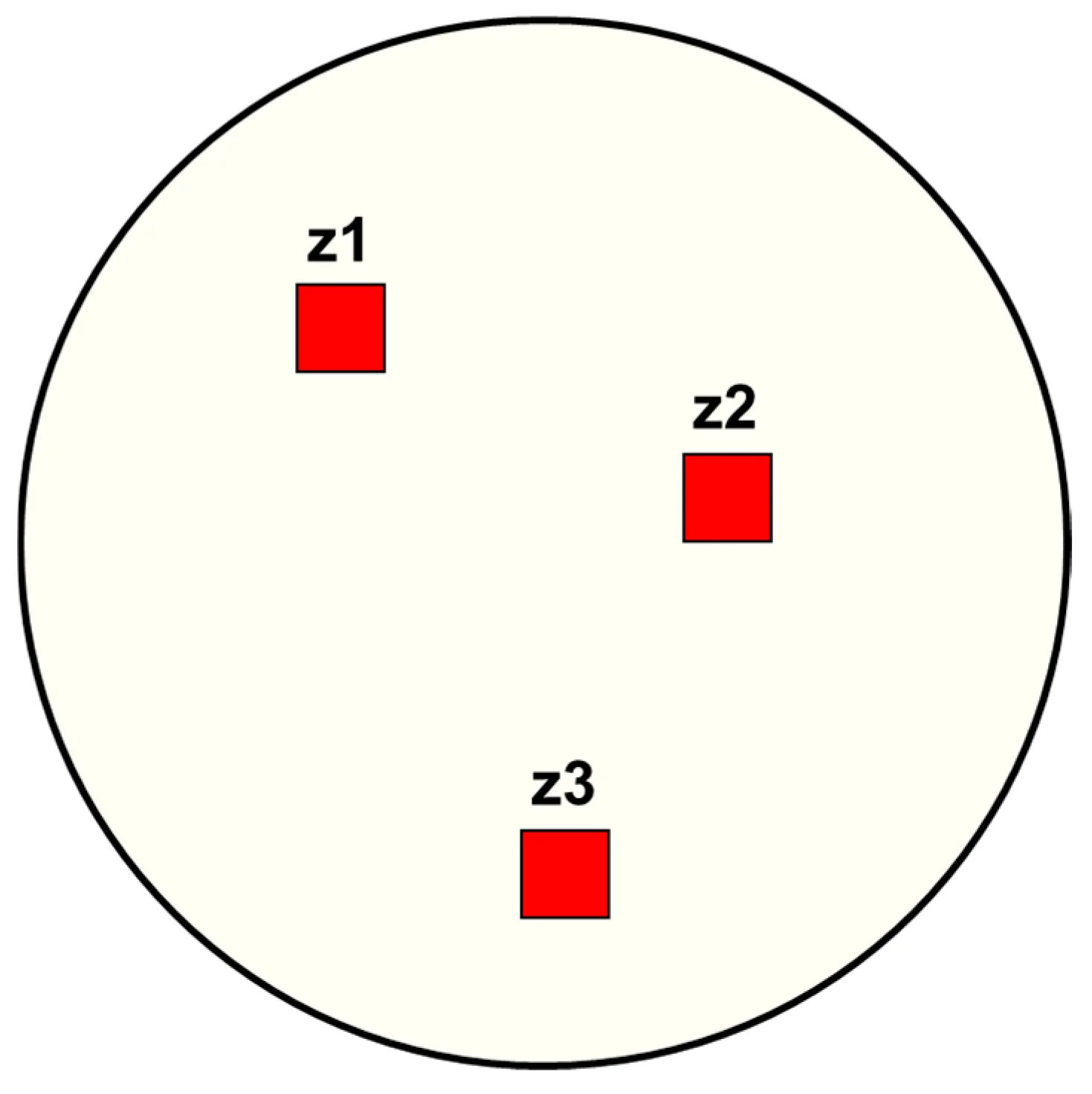
EDS-analyzed positions.

**Figure 5:**
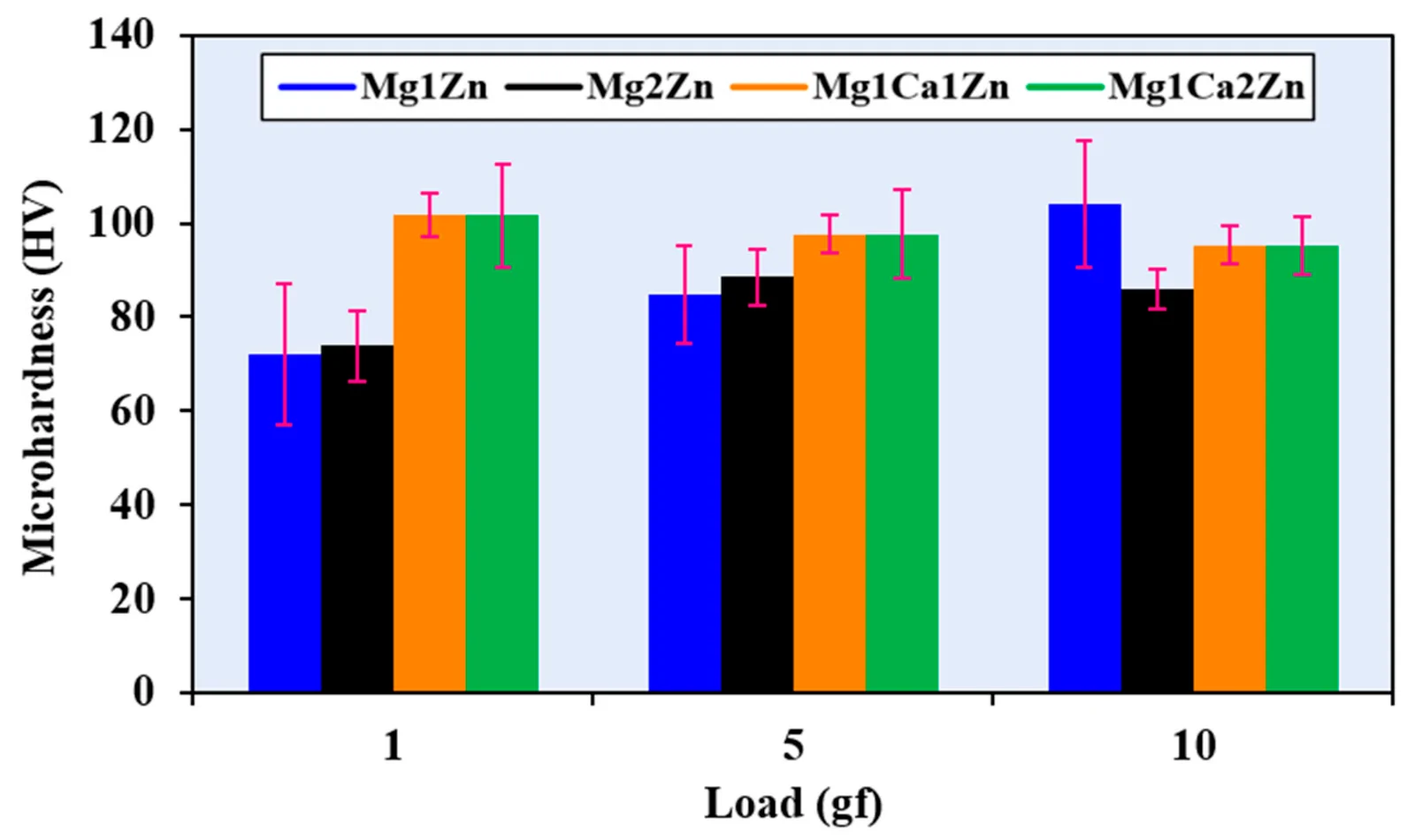
Average microhardness values for Mg1Zn, Mg2Zn, Mg1Ca1Zn, and Mg1Ca2Zn.

**Figure 6:**
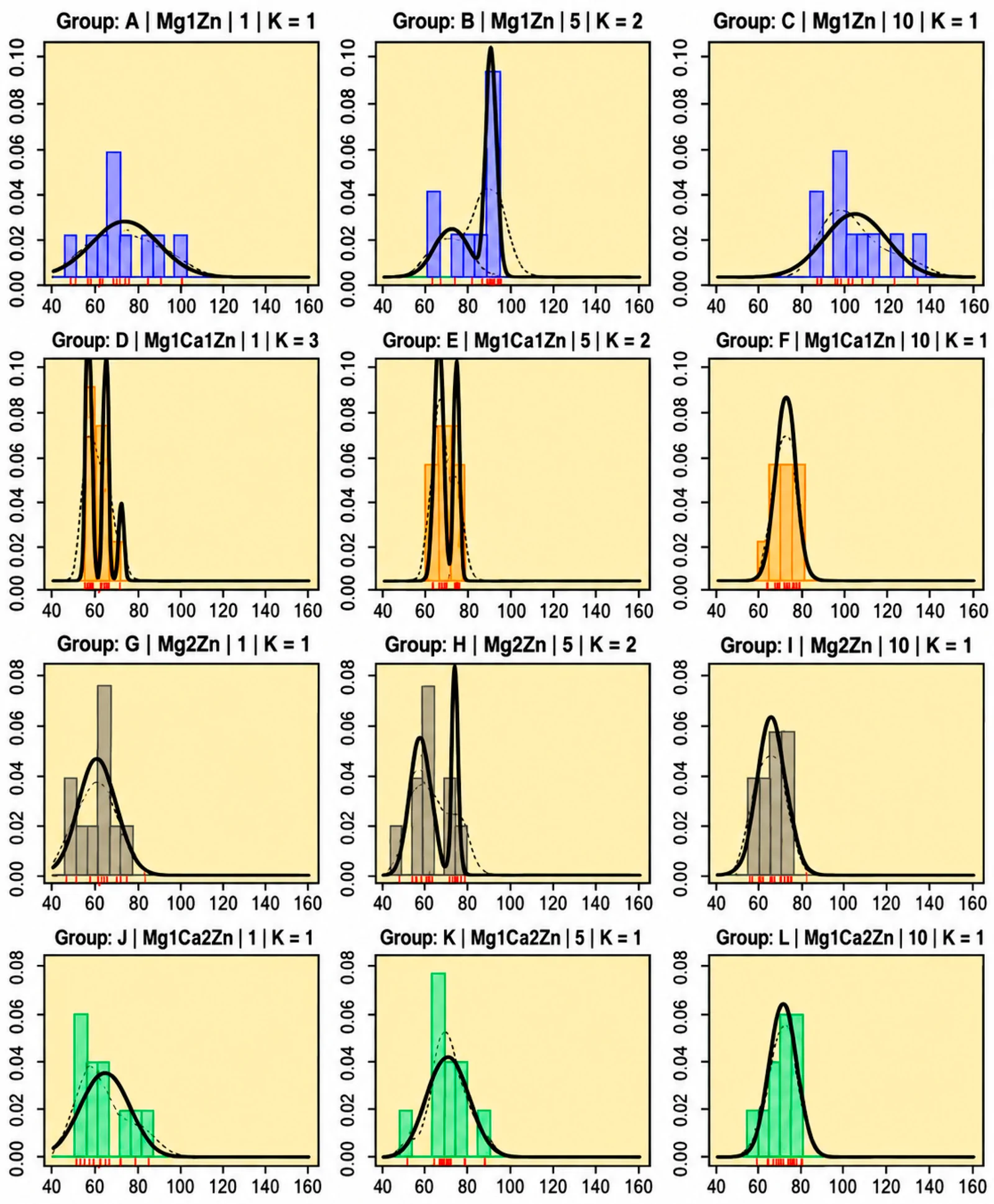
Microhardness values: histogram + Gaussian mixture per group (Alloy|Load).

**Figure 7:**
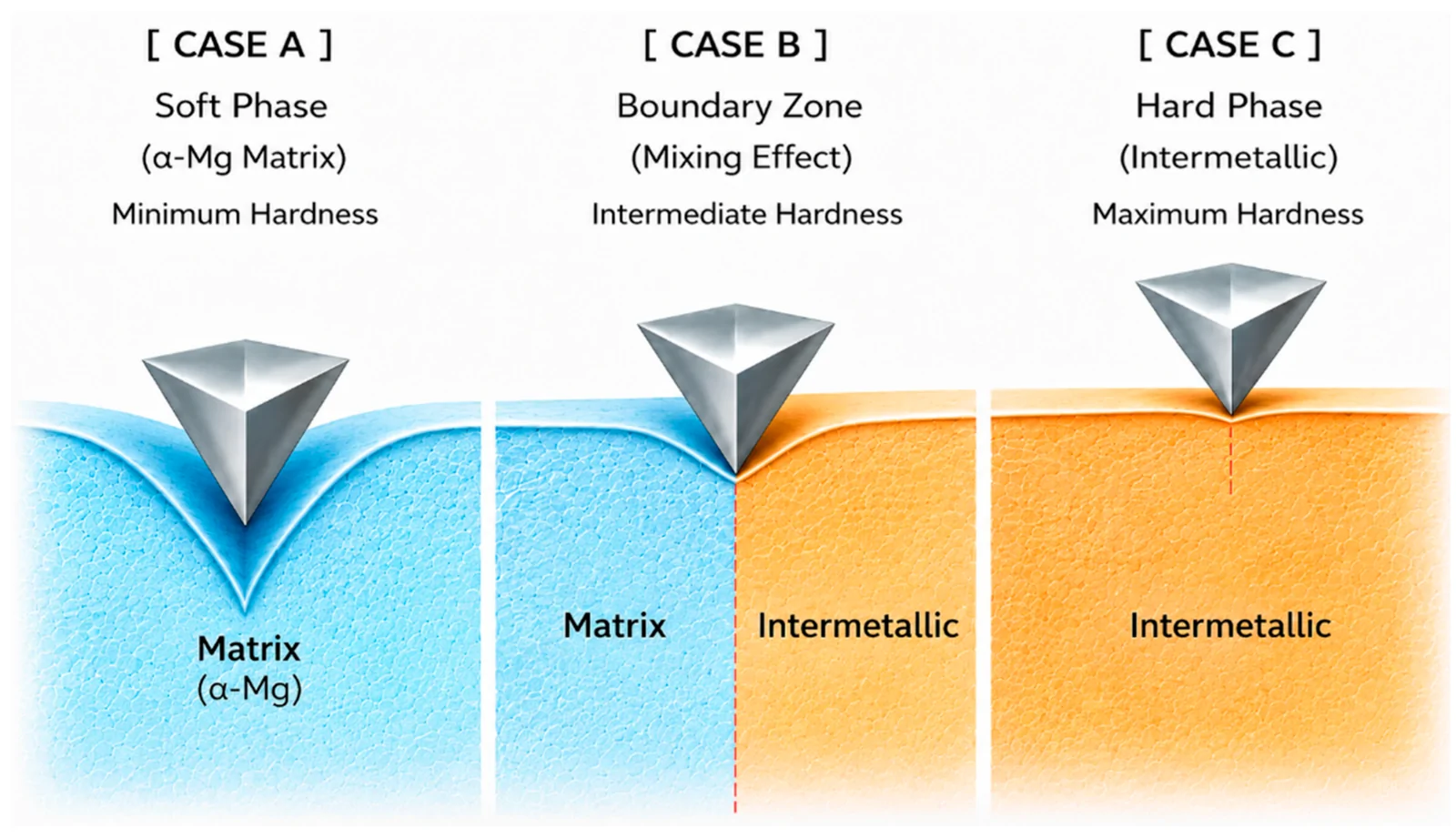
Schematic illustration of the indenter behavior on the alloy surface.

**Figure 8:**
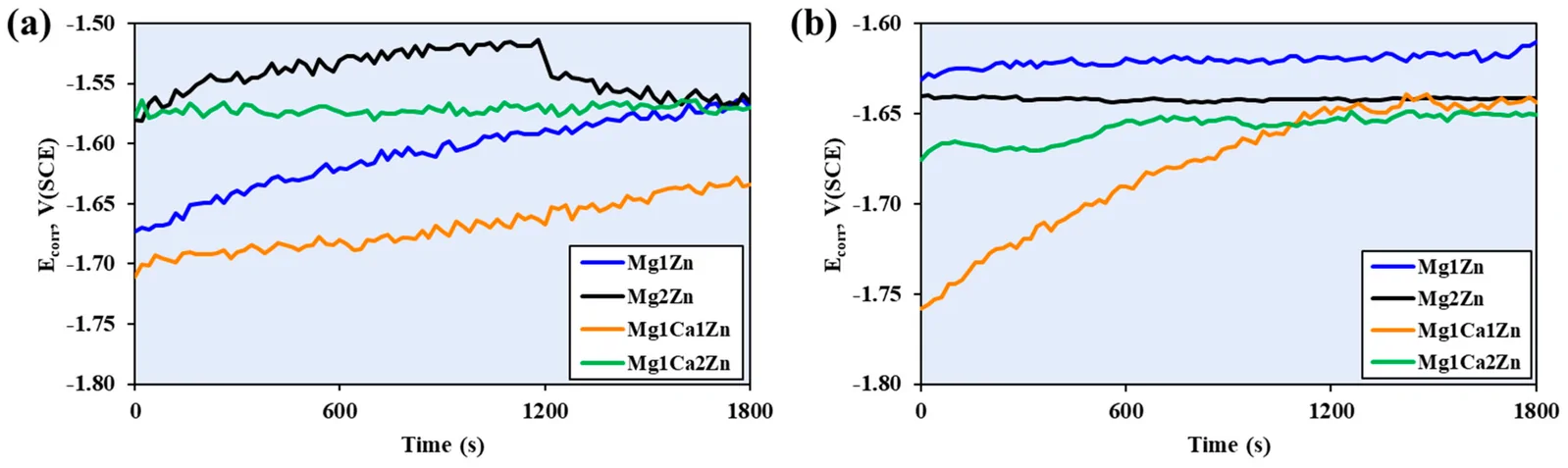
Corrosion potential after 30 min at: (**a**) 25 °C and (**b**) 40 °C.

**Figure 9:**
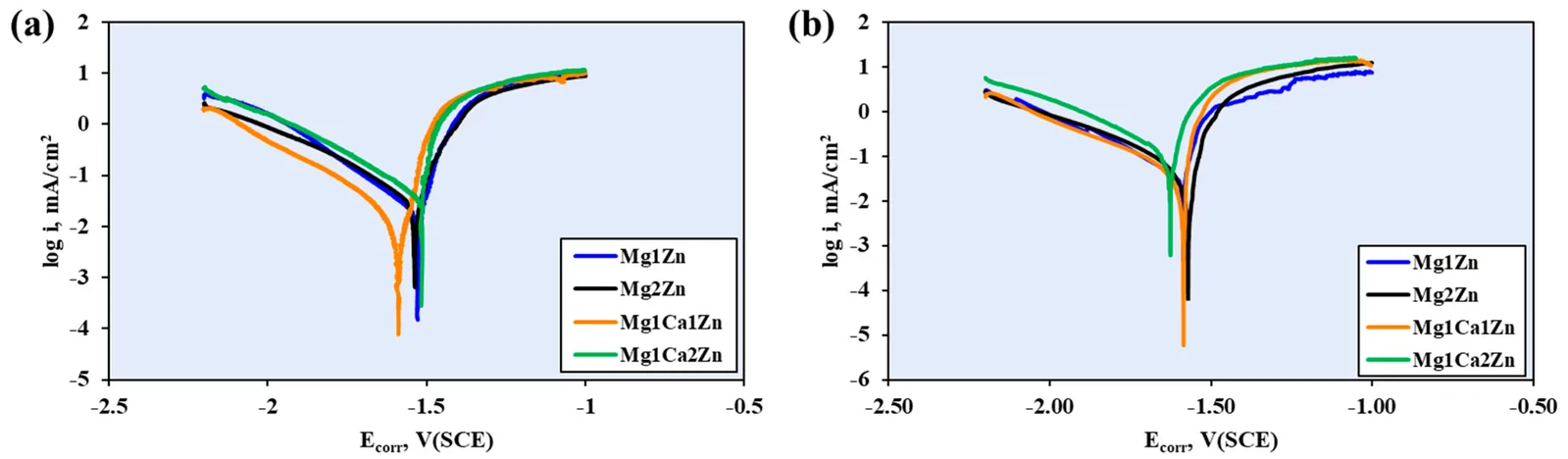
Potentiodynamic curves of the five samples at: (**a**) 25 °C and (**b**) 40 °C.

**Figure 10:**
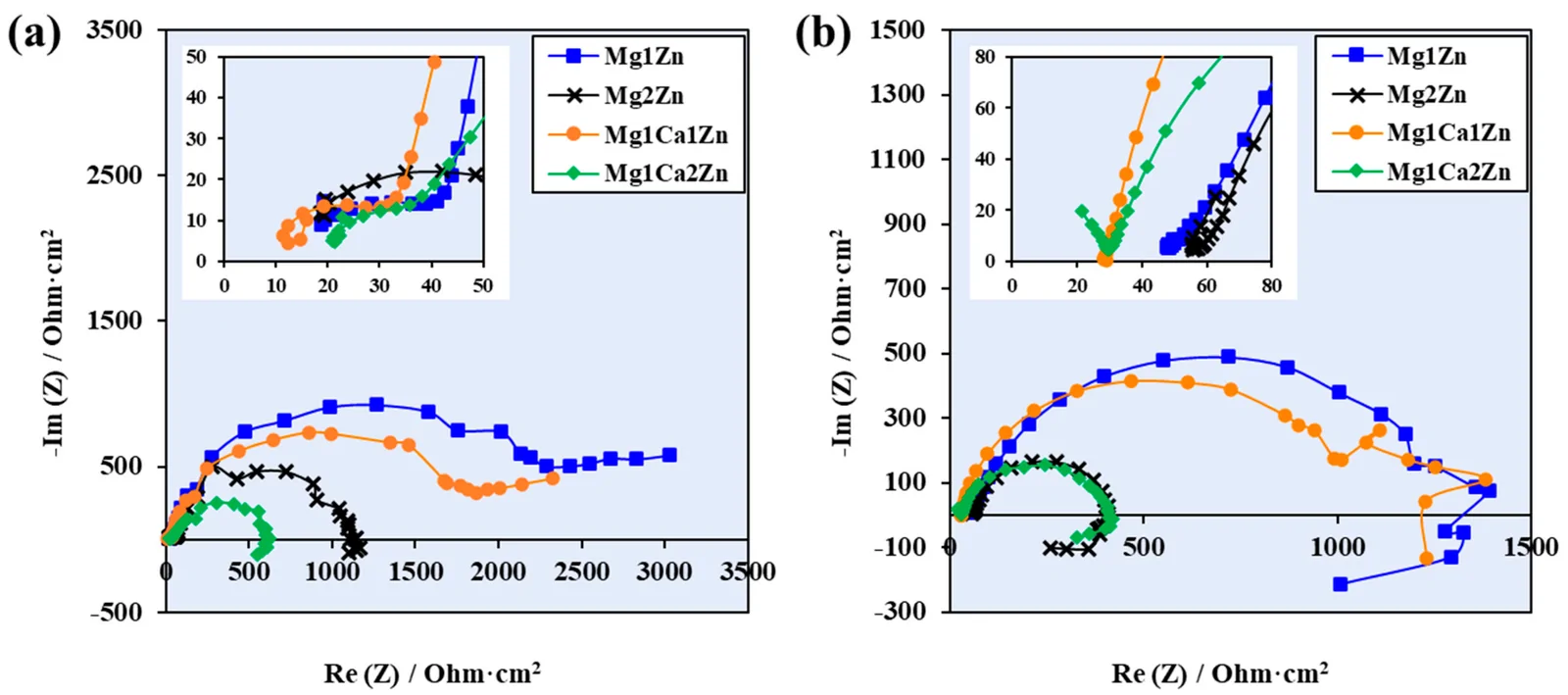
Nyquist diagrams of experimental data at: (**a**) 25 °C and (**b**) 40 °C.

**Figure 11:**
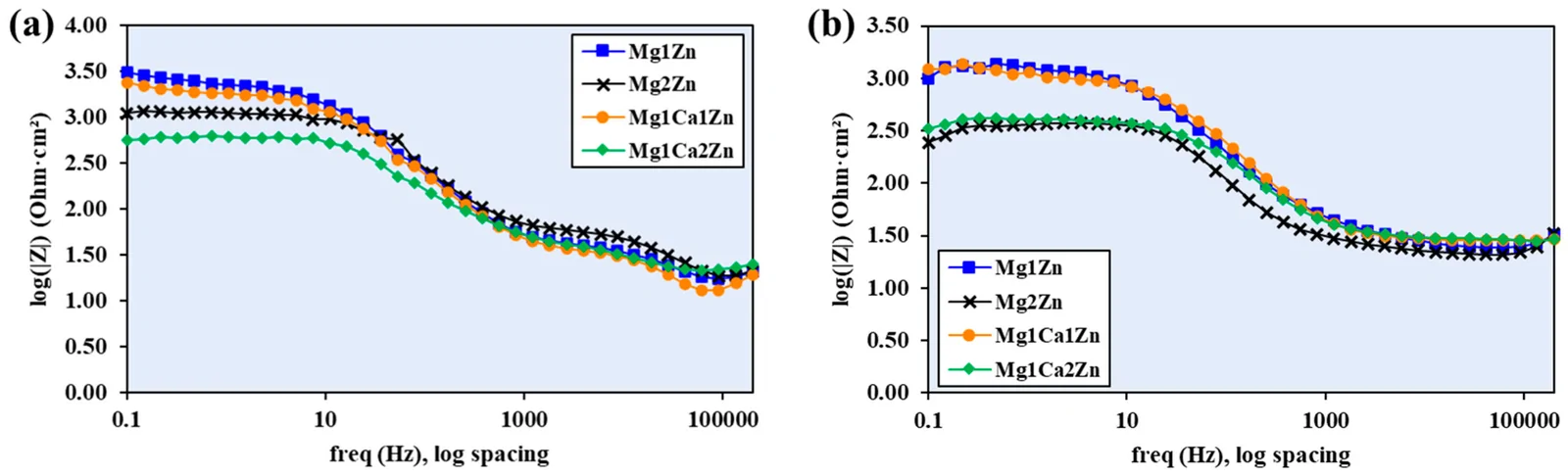
Bode-|Z| diagrams of experimental data at: (**a**) 25 °C and (**b**) 40 °C.

**Figure 12:**
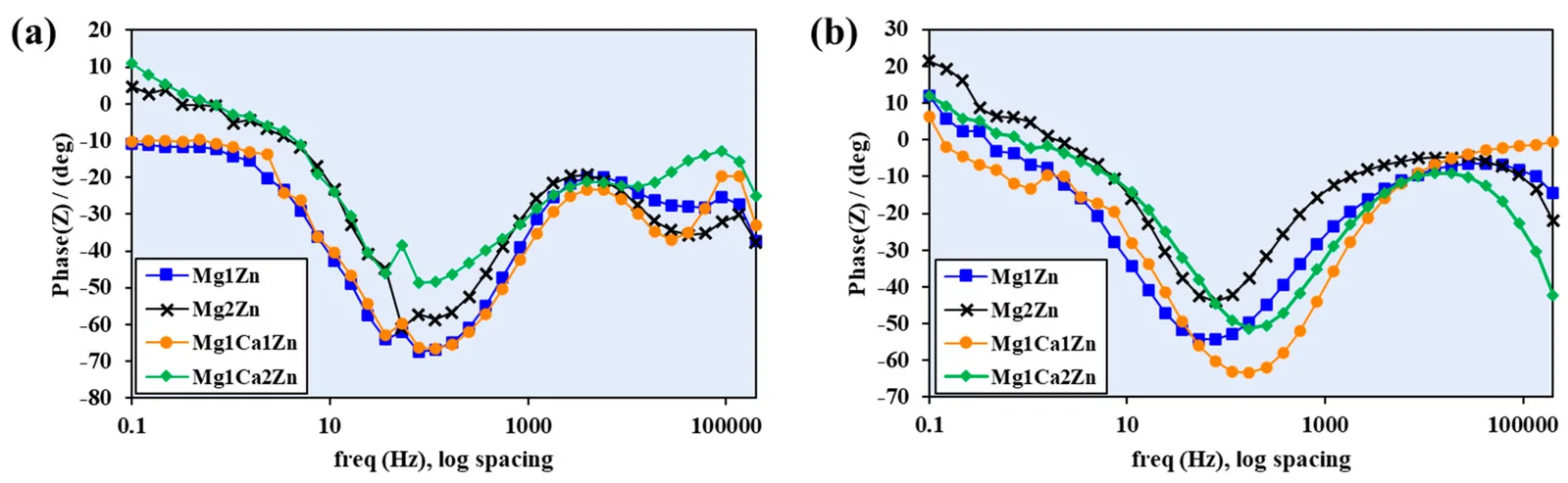
Bode-phase diagrams of experimental data at: (**a**) 25 °C and (**b**) 40 °C.

**Figure 13:**
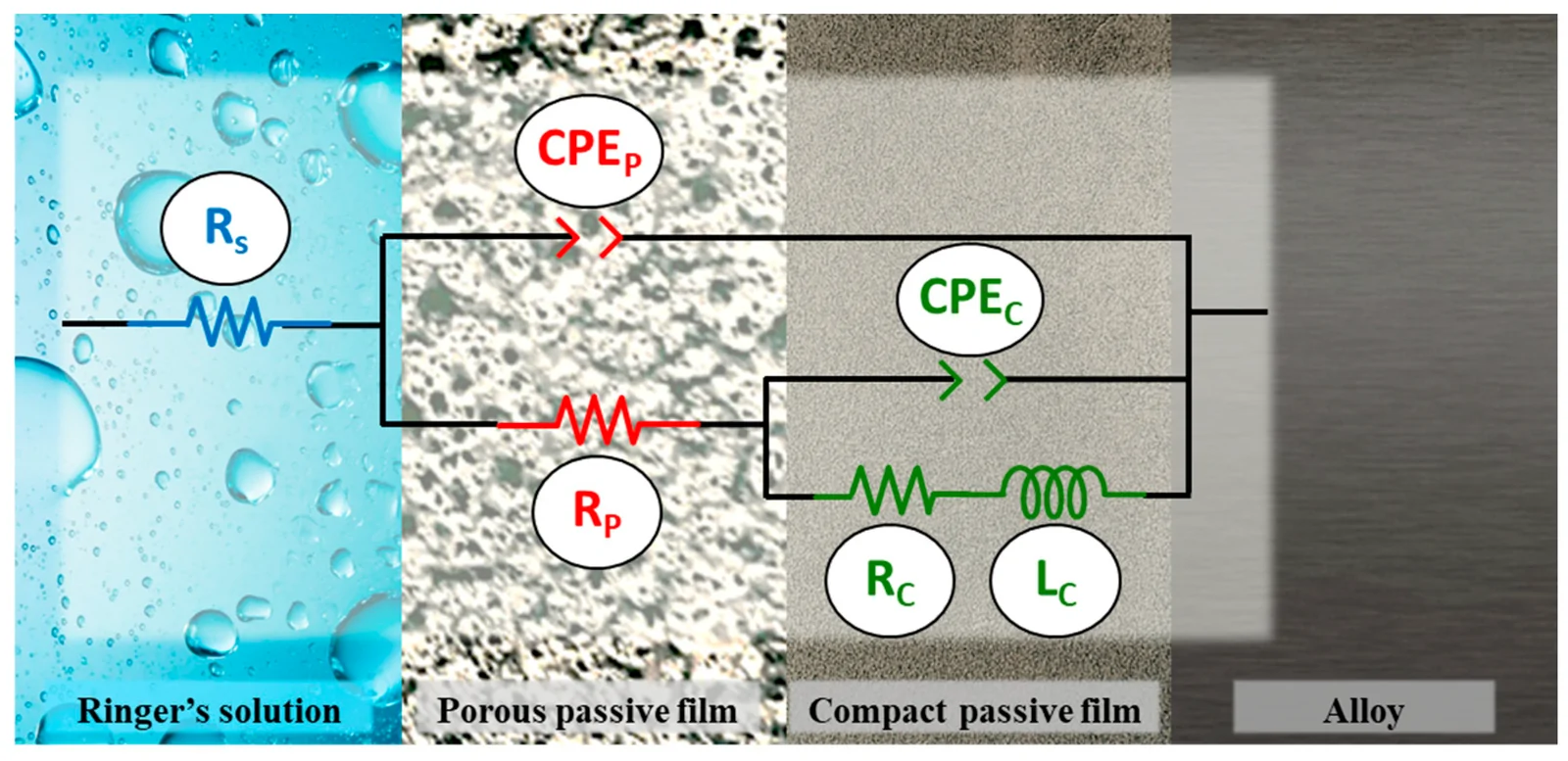
Electrical equivalent circuit R(CPE(R(CPE(RL)))).

**Table 1:** Nominal and EDS chemical analyses of the investigated alloys.

Samples	Elements	z1		z2		z3	
		wt.%	at.%	wt.%	at.%	wt.%	at.%
**Mg1Zn**	MgK	98.73	99.53	98.88	99.58	98.63	99.48
	ZnL	1.27	0.47	1.12	0.42	1.37	0.52
**Mg2Zn**	MgK	98.41	99.40	98.44	99.42	98.42	99.40
	ZnL	1.59	0.60	1.56	0.58	1.58	0.60
**Mg1Ca1Zn**	MgK	98.40	99.21	97.93	99.00	98.11	99.09
	CaK	0.79	0.48	0.93	0.57	0.86	0.52
	ZnK	0.81	0.31	1.14	0.43	1.03	0.39
**Mg1Ca2Zn**	MgK	96.21	98.28	96.52	98.43	96.44	98.37
	CaK	1.17	0.72	1.06	0.65	1.16	0.72
	ZnK	2.62	0.99	2.42	0.92	2.40	0.91

**Table 2:** Hardness values obtained subsequent to the application of the Gaussian mixture model (GMM).

Group	Alloy	Load	K	Comp	Weight	Mean	SD	BIC_K1	BIC_K2	BIC_K3
A	Mg1Zn	1	1	1	1	72.05	15.15	86.35	91.9	95.58
B	Mg1Zn	5	2	1	0.41	74.68	7.27	78.61	77.04	80.14
B	Mg1Zn	5	2	2	0.59	91.78	2.13	78.61	77.04	80.14
C	Mg1Zn	10	1	1	1	104.07	13.53	84.08	88.46	94.2
D	Mg1Ca1Zn	1	3	1	0.5	56.56	1	62.57	61.64	61.13
D	Mg1Ca1Zn	1	3	2	0.4	63.12	1	62.57	61.64	61.13
D	Mg1Ca1Zn	1	3	3	0.1	69.5	1	62.57	61.64	61.13
E	Mg1Ca1Zn	5	2	1	0.7	69.87	1.83	59.55	57.95	61.85
E	Mg1Ca1Zn	5	2	2	0.3	77.37	1	59.55	57.95	61.85
F	Mg1Ca1Zn	10	1	1	1	71.85	4.2	60.7	65.2	71.19
G	Mg2Zn	1	1	1	1	59.12	8.17	73.99	77.04	80.3
H	Mg2Zn	5	2	1	0.7	58.95	4.83	75.74	75.5	79.06
H	Mg2Zn	5	2	2	0.3	75.1	1.28	75.74	75.5	79.06
I	Mg2Zn	10	1	1	1	65.29	5.99	67.77	71.44	73.56
J	Mg1Ca2Zn	1	1	1	1	63.09	11.03	79.99	82.4	88.18
K	Mg1Ca2Zn	5	1	1	1	71.4	9.4	76.79	80.13	81.74
L	Mg1Ca2Zn	10	1	1	1	71.21	6.13	68.26	73.76	78.99

**Table 3:** Corrosion parameters of the samples studied.

Temperature	Samples	Equivalent Weight (g/eq)	Density (g/cm^3^)	Area (cm^2^)	E_corr_ (mV)	i_corr_ (µA/cm^2^)	β_c_ (mV)	β_a_ (mV)	CR (mm/Year)
25 °C	Mg1Zn	12.26	1.81	1.19	−1528	19	235	61	0.41
	Mg2Zn	12.35	1.82	1.2	−1536	32	244	73	0.59
	Mg1Ca1Zn	12.33	1.83	1.06	−1588	14	219	59	0.29
	Mg1Ca2Zn	12.45	1.88	1.04	−1517	40	275	34	0.84
40 °C	Mg1Zn	12.26	1.81	1.19	−1571	36	238	59	0.78
	Mg2Zn	12.35	1.82	1.2	−1571	37	210	52	0.69
	Mg1Ca1Zn	12.33	1.83	1.06	−1585	33	250	33	0.69
	Mg1Ca2Zn	12.45	1.88	1.04	−1626	106	266	62	2.21

**Table 4:** Results of fitting with the electrical equivalent circuit.

Temperature	Samples	Y_P_ (S·sec^n^/cm^2^)	n_P_	R_P_ (Ω·cm^2^)	Y_C_ (S·sec^n^/cm^2^)	n_C_	R_C_ (Ω·cm^2^)	L_c_ (Henri·cm^2^)	R_T_ (Ω·cm^2^)	Χ^2^
25 °C	Mg1Zn	6.8·10^−5^	0.50	59.4	3.9·10^−6^	1	3277	49.5	3336.4	4.4·10^−3^
	Mg2Zn	3.3·10^−6^	0.74	60.0	1.2·10^−5^	0.89	1073	2.3	1133	5.3·10^−3^
	Mg1Ca1Zn	3.8·10^−7^	1	23.8	2.4·10^−5^	0.81	1997	10	2020.8	6.6·10^−3^
	Mg1Ca2Zn	3.5·10^−7^	1	35.7	3.5·10^−5^	0.79	1238	4.42	1273.7	5.9·10^−3^
40 °C	Mg1Zn	1.7·10^−6^	0.61	43.4	3.1·10^−5^	0.79	1323	3.5	1366.4	3.8·10^−3^
	Mg2Zn	7.0·10^−9^	1	46.3	8.8·10^−5^	0.74	356.6	2.5	402.9	1.6·10^−2^
	Mg1Ca1Zn	1.7·10^−5^	0.91	345.2	2.1·10^−3^	0.56	948.9	284.7	1294.1	9.0·10^−4^
	Mg1Ca2Zn	3.710^−8^	1	19.7	5.4·10^−5^	0.76	398.7	1.2	418.4	5.9·10^−3^

## Data Availability

The original contributions presented in the study are included in the article, further inquiries can be directed to the corresponding author.
